# Evaluation of a One Health public health program based on minimum inputs to control *Taenia solium* in Madagascar

**DOI:** 10.1371/journal.pntd.0013624

**Published:** 2025-11-05

**Authors:** Diana Edithe Andria-Mananjara, Modestine Raliniaina, Mihaja Rakotoarinoro, José A. Nely, Nivohanitra Perle Razafindraibe, Noromanana Sylvia Ramiandrasoa, Bettelhein Ramahefasoa, Valisoa Claude Rakotoarison, Paul R. Torgerson, Eric Cardinale, Harena Rasamoelina-Andriamanivo, Glenn T. Edosoa, Agnès Fleury, Kabemba E. Mwape, Bernadette Abela, Marshall W. Lightowlers, Meritxell Donadeu

**Affiliations:** 1 National Center for Applied Research on Rural Development (FOFIFA), Antananarivo, Madagascar; 2 Ministry of Health of Madagascar, Antananarivo, Madagascar; 3 Directorate of Veterinary Services, Ministry of Agriculture and Livestock, Antananarivo, Madagascar; 4 CDC-One Health Indian Ocean-Indian Ocean Commission/SEGA-One Health Network, Quatre Bornes, Mauritius; 5 Consultant, Antananarivo, Madagascar; 6 Section of Epidemiology, Vetsuisse Faculty, University of Zürich, Zürich, Switzerland; 7 Anses, National Health Agency, Paris, France; 8 World Health Organization Country Office Antananarivo, Madagascar; 9 Departamento de Medicina Genómica y Toxicología Ambiental, Instituto de Investigaciones Biomédicas, Universidad Nacional Autónoma de México, Ciudad de México, México; 10 Clínica de neurocisticercosis, Instituto Nacional de Neurología y Neurocirugía, Ciudad de México, México; 11 Department of Clinical Studies, School of Veterinary Medicine, University of Zambia, Lusaka, Zambia; 12 Malaria and Neglected Tropical Diseases Department, World Health Organization, Geneva, Switzerland; 13 Department of Biosciences, Melbourne Veterinary School, University of Melbourne, Werribee, Victoria, Australia; 14 Initiative for Neglected Animal Diseases (INAND), Midrand, South Africa; Hospital General Dr Manuel Gea Gonzalez, MEXICO

## Abstract

Cysticercosis in humans caused by the parasite *Taenia solium* is one of the World Health Organization’s Neglected Tropical Diseases. The parasite is transmitted between the human host and pigs. Efforts to prevent the disease have relied mainly on treatment of people with anthelmintics. However, to date, there is no practical and effective control method that has been delivered as a public health program. Here we describe a large-scale, minimum inputs *T. solium* control program implemented as a public health program in Madagascar. Initially all pigs were vaccinated for porcine cysticercosis and medicated with oxfendazole, after which only young piglets and pigs imported into the program area were targeted for interventions. After piglet interventions were in place and on-going, a single mass drug administration (MDA) was delivered to the human population with a taeniacide. The outcomes were assessed one year after the human treatment, by comparing pre-and post-intervention levels of porcine cysticercosis caused by *T. solium* and human *T. solium* taeniasis. Over a twenty-two-month period, 96,735 pig vaccinations and oxfendazole medications were delivered and during the MDA, 117,216 people received taeniacide. Ninety percent of the pig population were receiving vaccination and medication at the end of the intervention period. Coverage of the eligible human population by the MDA was 62.5%. Prior to the intervention 30.8% of slaughter-age pigs had viable *T. solium* infection, reduced to 8% after the program. Human taeniasis was found to be 1.25% prior to the MDA and 0.6% one year after the MDA. The program successfully demonstrated effective control of *T. solium* transmission to pigs using minimum inputs and delivered as a public health program. Sustained control and expansion of the program could potentially lead to the elimination of the disease being a public health problem in Madagascar.

## Author summary

Cysticercosis caused by the cestode parasite *Taenia solium* is one of the World Health Organization’s neglected tropical diseases. We implemented One Health cysticercosis control as a public health program in a highly endemic region of Madagascar. The program was designed to require minimum inputs while achieving maximum impacts on control within a short period of time. Pigs were vaccinated with the TSOL18 vaccine and simultaneously treated with oxfendazole. Over a 2-year period, piglets 2–3 months of age were treated. After pig vaccination and medications where established, the human population received a single treatment with a taeniacide. The program was evaluated by assessment of porcine cysticercosis and human taeniasis before and 12 months after the human mass drug administration. More than 95,000 pig vaccination and medications were delivered and 117,000 persons received taeniacide. The intervention significantly reduced the prevalence of cysticercosis in slaughter-age pigs and entirely eliminated viable porcine cysticercosis in animals that had received two vaccinations and medications. Continuation and expansion of the program could have the potential to eliminate *T. solium* from the program area, or even from the entire country.

## Introduction

Neurocysticercosis (NCC) is an infection of the central nervous system caused by the larval stage of a zoonotic parasite, *Taenia solium* [1]. It is prevalent in many parts of the world where risk factors for the parasite’s life cycle are present, such as poverty, lack of hygiene and sanitation, and the presence of free-roaming pigs [1]. As a result, many low- and middle-income countries, including Madagascar, are endemic to *T. solium* [2,3], and NCC is a leading cause of epilepsy in the population [4].

NCC is considered a preventable and potentially eradicable neglected tropical disease [5]. So far, attempts to control transmission of *T. solium* have relied mainly on preventive chemotherapy in humans using either praziquantel or niclosamide, at times incorporating other interventions as well [6–9]. Another tool available for the control of *T. solium* is the combined use of the TSOL18 (Cysvax) porcine cysticercosis vaccine together with oxfendazole medication in pigs. The vaccine has proved to be safe and effective; two doses separated by approximately 4 weeks induce a protective response. Vaccination in combination with oxfendazole medication has been found to eliminate parasite transmission by the treated pigs in field trials undertaken in Nepal, Cameroon, and Uganda [10–12]. Treatment of 3–4-month-old pigs with oxfendazole alone does not prevent subsequent infection of the animals and the presence of mature, viable cysticerci at slaughter age [11].

One Health research projects based on mass drug administration (MDA) and/or targeted chemotherapy in combination with other One Health measures (mainly pig vaccination and/or oxfendazole medication) have achieved a reduction in *T. solium* transmission [13–17]. However, the approaches used have either had a limited impact on transmission or were resource intensive and unlikely to be implemented as public health programs. There is currently no effective model for the control of the parasite that has been implemented and has potential for adoption as a national public health program. Mathematical modelling of *T. solium* transmission has indicated that an approach involving treatment of the pig population by vaccination and oxfendazole medication, together with human treatment by MDA would be the most efficient in achieving elimination of parasite transmission [18,19].

Here we sought to evaluate the effectiveness of a *T. solium* control strategy in an endemic region of Madagascar. Within the context of many research projects and the Malagasy settings, the control strategy adopted was considered to involve minimum inputs, and had potential to lead to a substantial and potentially sustainable reduction in parasite transmission. The program was capable of being delivered as a public health program by the Veterinary Services of the Ministry of Livestock and the Ministry of Public Health of Madagascar. Principal components of the program involved TSOL18 vaccination and oxfendazole medication of young piglets, and a single round of taeniacide MDA in the human population after pig vaccination and medication had been well established. Our intention was to undertake the MDA after the pig population no longer presented a risk for the establishment of new cases of taeniasis. The primary outcome was the effectiveness of our *T. solium* control strategy, which was assessed by comparing pre- and post-intervention levels of porcine cysticercosis and human *T. solium* taeniasis. This included:

1. Assessments of pre and post intervention prevalences of porcine cysticercosis,
2. Assessments of pre and post intervention abundance of porcine cysticercosis, and
3. Assessments of pre and post intervention prevalences of human taeniasis.

The hypothesis to be tested was that a combination of pig vaccination and medication, delivered twice to each animal, plus a single treatment of the human population with a taeniacide, will significantly reduce transmission of *T. solium* as determined by changes in the level of porcine cysticercosis and human *T. solium* taeniasis.

## Methods

### Ethics statement

Intervention and assessment protocols were designed and implemented in compliance with ethical approvals for the study granted by the Madagascar Ministry for Public Health Ethics Committee for Biomedical Research No. 088-MSANP/SG/AMM/CERBM and by the Malagasy National Animal Ethics Committee, No. 001-21/CENA.

### Study area and timeline

**Fig 1:**
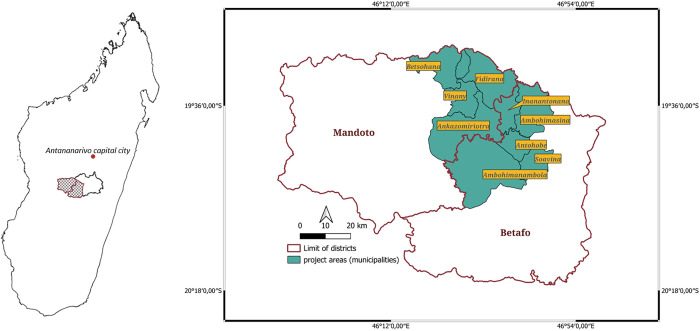
*Taenia solium* One Health control program implementation areas in Madagascar. Map generated using QGIS 3.30.2 (QGIS Development team in https://www.qgis.org/). Map boundary data was derived from the Humanitarian Data Exchange (HDX) (https://data.humdata.org/dataset/cod-ab-mdg), managed by the United Nations Office for the Coordination of Humanitarian Affairs (OCHA) and made available under a Creative Commons Attribution for Intergovernmental Organisations license.

The control program was implemented from August 2021 to August 2023 in a single contiguous area in central Madagascar comprising nine municipalities in two districts of Vakinankaratra region, Betafo and Mandoto (Fig 1). The area comprises approximately 420 villages (hamlets) spread over 84 fokontany (the lowest administrative unit), 205,197 inhabitants in 45,539 households [20] and an estimated 27,000 pigs. The most important economic activity among 93% of the population is mixed cropping with livestock farming [20]. Pig farming is one the major livestock activities of households. It is used as a means of livelihood and a source of savings for small scale farmers [21]. The program area was selected because the prevalence of human cysticercosis in the region was relatively high [22]. It is also one of the main suppliers of pigs for Antsirabe (the largest city in the region) and the capital of Madagascar, Antananarivo [23], and cases of porcine cysticercosis were seen frequently in Vakinankaratra compared to other regions of the central highlands [24].

### Program design and rationale

Interventions to interrupt *T. solium* transmission were undertaken in the two obligate hosts of the parasite, pigs and humans. Implementations were designed as One Health interdependent actions and included training of actors, advocacy and social mobilization to maximize the involvement of the relevant stakeholders.

The timings of the interventions were a key component of the program design. There are no data on susceptibility of humans to re-infection with *T. solium* adult tapeworms however animals are susceptible to reinfection with a variety of adult taeniid species, with either little or no evidence of immunity to the adult tapeworm stage [25,26]. This being the case, it seems likely that, after receiving the MDA, humans could get infected or re-infected with *T. solium* adult worms after ingesting contaminated pork. For this reason, our first step was to reduce the risk of infection/re-infection with adult tapeworms in humans by reducing the prevalence of the disease in the pigs by vaccinating and medicating the entire eligible pig population. The human MDA was planned to be conducted only after the risk was minimised through vaccination and medication of the pig population. However, because of the likely imperfect coverage of the MDA, and the potential risk of tapeworm eggs surviving in the environment, the vaccination and medication of piglets and incoming pigs continued for a year after the MDA, after which assessments were undertaken of the impact of the program on porcine cysticercosis and human *T. solium* taeniasis.

While the implementation was based on activities conducted as a public health program (therefore implemented by existing staff linked to the Ministry of Public Health or the Veterinary Services), the evaluation was conducted as a research project so as to obtain accurate measures of the impact of the intervention on *T. solium* transmission. The local coordination and supervision were provided by the National Center for Applied Research on Rural Development (FOFIFA).

### Pig intervention

Intervention activities in pigs were undertaken between October 2021 and August 2023. Initially a census of the pig population in the program areas was conducted to determine the number of pigs. The census was conducted by the chiefs of the fokontany and their teams who visited each household in the area, and was coordinated by FOFIFA. Subsequently, pigs in the area were vaccinated with the TSOL18 vaccine (Cysvax from Indian Immunologicals Limited) and, simultaneously, received an oral treatment with oxfendazole (Oxfenvet from Indian Immunologicals Limited). The vaccines and drugs were purchased with funds from the program sponsor. Interventions in the pig population were undertaken by the staff of the Ministry of Livestock through the Veterinary Services Directorate, the sanitary veterinarians and their animal health agents (vaccinators). The vaccinators already had a contractual relation with the veterinarians, but they received additional income from funds provided through the Ministry from the program sponsor.

**Fig 2:**
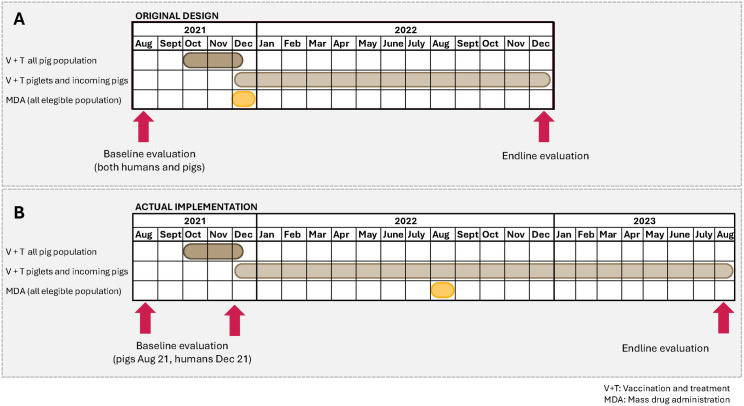
One Health model for the control of *Taenia solium* in Madagascar. Fig 2A shows the original design, and Fig 2B shows the actual implementation after the MDA had to be delayed.

At the start of the program, the intervention targeted all the pigs in the population. Inclusion criteria were animals ≥ 2 months of age, showing no obvious signs of sickness and not pregnant or lactating (those animals were vaccinated and medicated in subsequent visits, if they complied with the eligibility criteria). The animals received two immunizations, approximately one month apart, with the TSOL18 vaccine and, at the time of the second immunization they also received treatment with oxfendazole at 30 mg/kg. Subsequently, throughout the program, newborn piglets (at ≥ 2 months of age) and any pigs imported into the program area received both TSOL18 vaccination and oxfendazole medication twice, at least 1 month apart (Fig 2). At the time of the first vaccination and medication, the animals were tagged with a button tag in one ear. At the time of the second vaccination and medication, the button tag was marked with indelible pen. There were no unique identifiers used to identify individual animals. Vaccinators visited the villages approximately on a monthly basis, and farmers having newborn piglets or imported animals were identified by the farmer themselves, the Chief of the fokontany or neighbouring farmers. The program aimed to identify and treat any animals which did not initially receive treatment for some reason, during the following monthly visits to the farms. The interval between vaccinations is not critical and intervals of 2–4 months between primary and secondary immunization provide as good, or better, response to the vaccine in comparison to animals receiving vaccinations 4 weeks apart [27]. The strategy was efficient in targeting piglets but less reliable for older animals as they were often roaming in the fields and difficult to reach, this being a key reason why older pigs were only targeted at the start of the program to accelerate the coverage, and the main strategy was based on piglet vaccination.

The vaccination and medication status of the animals treated, at both their first and second treatments, were recorded by the vaccinators although individual animals were not identified. Untreated pigs owned by farmers that agreed to participate in the program were always treated, and hence recorded as treated once. Pigs identified by the vaccinators as having previously received treatment were always treated again and recorded as being twice treated. The on-going treatment status of the entire pig population was not measured precisely, since every farm was not visited every month, which would have required greater resources and logistics than were available. From August 2022, it was decided to use a proxy measure, by asking the vaccinators to record the vaccination and medication status of the pigs they meet during their monthly vaccinations. Because the vaccinators were only going to the households where they knew there were pigs to be treated (as learned from previous visits, the Chief of the fokontany or other farmers), they were not capturing the total pig population as they were not going to households where, for example, all the pigs were already treated. This is an imperfect measure as pigs could have been missed, but it helped to identify areas where the coverage (the proportion of pigs that received two vaccinations and medications among all encountered and eligible pigs, hereinafter referred to as coverage estimate) was below 75% and where additional awareness efforts were required, such as additional radio campaigns or visits to the communities.

Vaccinators were required to maintain strict biosecurity measures to prevent the spread of pig diseases, in particular African and Classical swine fevers. Disinfection of boots, overalls, lassoes, and other equipment with chlorine (Virokil, Agrivet Antananarivo, Madagascar) was mandatory before entering and after leaving a farm. Vaccine was administered using single-use needles, and both vaccine and oxfendazole were administered using disposable syringes. Used items were stored in separate bags and transported to the veterinarians, who in turn disposed of it in an appropriate manner.

### Human intervention

A single MDA was undertaken in the human population once the intervention in the pig population was anticipated to have been fully established (August 2022, Fig 2). The MDA was initially planned for December 2021, but due to adverse events with a child receiving praziquantel during a MDA program for schistosomiasis in a nearby district, the Ministry of Public Health delayed all the MDA interventions until further training and preventive measures, such as the use of an alternative drug (niclosamide) and early detection and management of any adverse events, were put in place [28]. For that reason, the MDA was not undertaken until August 2022. The Ministry of Public Health through its communicable disease control department and the local medical staff (regional and district responsible for neglected tropical diseases, chiefs of health centers and community health agents), undertook the MDA campaign in all the eligible population over 5 years of age. The main drug used was praziquantel (Cesol from Merck or Biltricide from Bayer donated through the WHO). As MDA with praziquantel was already part of the Ministry of Public Health’s annual program for treatment of schistosomiasis (co-endemic with *T. solium* in this region), the MDA was conducted by the Ministry of Public Health in synergy with their MDA campaign against schistosomiasis. Children between 5 and 15 years of age targeted by the schistosomiasis program, received praziquantel at 40mg/kg. Additionally, the children in Betafo district, also received mebendazole (500mg, Vermox, Johnson & Johnson) as part of the Ministry of Public Health program to control soil-transmitted helminths. People 15 years old and over, were treated with praziquantel at 10mg/kg as a taeniacide. Prior to the MDA, a series of training and activities were undertaken to prevent and minimise neurological adverse events [28]. All people over 5 years of age were eligible for treatment except for pregnant women, women breastfeeding children 3 months or younger and those with symptoms or signs compatible with NCC such as seizures and epilepsy or chronic headache. The coverage of the population was calculated based on the number of people who received treatment during the MDA and the number of eligible targeted population. Those exhibiting symptoms or signs compatible with NCC were treated with niclosamide (Yomesan, Bayer) at a dose of 1g for children 5 or 6 years of age, and 2g for children over 6 years and adults.

Active and passive surveillance were conducted to monitor and quickly identify and manage any possible adverse events in people who had participated in the MDA [28]. Local staff were trained, and appropriate medicines were made available at health centers to deal with side effects, such as neurological events, drug allergies, etc.

### Training and monitoring

Prior to the interventions, training was undertaken for those involved in activities related to animals or humans.

Veterinarians and vaccinators were trained by the Veterinary Services of the Ministry of Livestock in vaccination and medication procedures, maintenance of cold chain, identification of vaccinated and treated animals, oral drug medication, coverage data collection, and biosecurity measures. Messages to transmit to farmers who may have been initially reluctant to take part of the campaign were also covered during the training. During the pig treatment campaign, vaccinators were supervised and audited by local veterinarians with monitoring by FOFIFA staff.

The coverage estimate for pig interventions was determined from monthly reports from the vaccinators (proxy data, as detailed above) and was calculated, separately, based on the number of pigs that received one dose or two treatments with vaccine and medication, and the total number of eligible pigs in the area.

Those involved in the human intervention received cascade training provided by the Ministry of Health, with the support of the project neurologist. Modules covered a refresher on the disease complex, standard operating procedures for the MDA (which is already part of the annual Ministry program), key messages to increase participation, data collection for the coverage estimate, active surveillance of NCC cases and potential adverse reactions, and management of adverse events [28]. Monitoring and supervision of activities were undertaken by the Ministry of Health senior staff pre- and post-MDA campaign.

### Community sensitization

Social mobilization was a key component for this program. The local authorities were the first to be involved, and with their cooperation awareness campaigns were undertaken to facilitate access to beneficiaries and maximize their participation either in the animal or human interventions. Traditional singers were recruited to compose awareness-raising songs related to the fight against *T. solium* and to perform the songs in local tours in the municipalities covered by the program. In addition, radio spots were strategically broadcasted on local channels. The key messages for these two types of sensitizations were related to the parasite’s transmission, the importance of the fight against *T. solium*, encouragement of farmers to participate in the pig vaccination and medication program, encouragement of people to participate in the MDA, and the clarification of any negative rumours circulating about the interventions.

Posters were produced in local language informing the farmers of the next date pig treatments were to be delivered and to encourage participation. These were posted in the villages and used at community meetings. The program used the WHO/WOAH/FAO *T. solium* poster on the cycle of the parasite and the possible ways of control [29]. Other posters on the identification and management of adverse events were also prepared and used during the MDA [28].

### Data collection

Data from all interventions were recorded through paper-based forms. For pig interventions, data were recorded monthly by vaccinators and focused on the number of animals in each farm, the number of vaccinated and medicated animals according to the number of treatments provided, and the number of non-treated animals per category (< 2 months, pregnant sow, lactating sow). Ear tags allowed identification of an animal’s treatment status (once or twice vaccinated and medicated), however individual animals were not identifiable. Local supervising veterinarians collected all data recorded and made a monthly report for the senior staff.

For the MDA, data collected related to the demographic characteristic of the person who received the drug, the presence of signs compatible with NCC, the drug administered, adverse events, the clinical signs linked to the adverse event, and the treatment received.

### Program evaluation

The program was evaluated by determining changes in pre- and post-intervention levels of viable porcine cysticercosis infections in the pig population, pre- and post-intervention abundance (intensity) of porcine cysticercosis in untreated pigs, and pre- and post-intervention levels of human *T. solium* taeniasis. The objective was always to conduct the endline evaluation one year after the MDA; because the MDA was delayed, the final evaluation was also delayed. Baseline and endline evaluations were conducted, respectively, in August 2021 and August 2023 for porcine cysticercosis. Evaluations for *T. solium* taeniasis were carried in October 2021 (repeated in December 2021) and August 2023.

#### Sample size calculation and sampling strategies.

Porcine cysticercosis was determined by detailed necropsy examination on slaughter-age pigs selected randomly and purchased from the study area population. The sample size for necropsy assessment (104 animals) was calculated based on the expected initial necropsy prevalence of 15%, from a population of 45,000 pigs (as per the latest official census) distributed in 20 villages, each ranging from 350 animals upwards per village, with the samples assumed to be aggregated. Samples size was calculated with a power of 90% and 99% confidence interval. Calculations were done in R (version 3.6.0) via Monte-Carlo simulations. For *a priori* sample size estimation, it was assumed that infection in pigs was over dispersed. Thus, the sample size estimate was adjusted for clustering by use of the negative binomial distribution to first estimate the expected reduction in total cysts then converted to prevalence (from the expected zeros in the simulated cyst abundances) to run the power calculation using the pwr package in R. Specificity (by necropsy) was assumed to be 100%, whilst sensitivity 71%. Effect size for pigs was assumed to be a reduction in prevalence from 15% to less than 2%.

At baseline, 30% of the fokontany were randomly selected for sampling (i.e., 26 fokontany), and the same fokontany were used for the final evaluation. Prior to baseline and final evaluation, a census of the slaughter age pigs was conducted (at baseline by the chief of fokontany and their teams, and at endline by the vaccinators), and from those lists, the animals were randomly selected by using the random function in Microsoft Excel 2013. The number of pigs selected per fokontany, was proportional to the number of pigs. For the endline assessment, animals were identified as having received two vaccination and medication treatments, only one vaccination and medication treatment, or untreated. From those that were either fully treated (two vaccinations and medications) or untreated, 104 animals were selected for necropsy according to the same criteria used in the baseline evaluation [30]. As there were 34 animals non-vaccinated selected for the final evaluation, an additional 16 slaughter-age pigs were selected purposively from those in the population that had not received any vaccination or oxfendazole, to have a total of 50 non-vaccinated or medicated pigs so as to improve the accuracy of evaluations of the abundance (intensity) of infection in untreated pigs.

A sample size of 960 people to evaluate taeniasis was calculated from an estimated target population of 190,000, which would detect a reduction in prevalence from 1.8% to <0.1%, with a power of 90% and confidence level of 99%. Calculations were done in R via Monte-Carlo simulations. The first evaluation conducted in October 2021 found four positive samples out of 960, which was not congruent with the high level of infection in pigs determined during the baseline necropsies. Therefore, the sampling design was adjusted, and a new purposive sampling was conducted, targeting specifically villages in which roaming pigs and deficient sanitation were present, but randomly selecting the people within those villages. For the new baseline and the final evaluation, fecal samples from 960 people were randomly sampled (according to the size of the pig population) from 30% (n = 26) of the fokontany, which were randomly selected as a separate exercise from the fokontany selected for the pig evaluation. The number of samples per village was also proportional to the number of pigs in the village, and not to the number of people, as due to the cycle of the parasite, it is less likely that there is active transmission in the larger towns in which there are fewer (or no) roaming pigs and better sanitation.

#### Diagnostic methods.

Pig necropsies were undertaken as previously described [30]. Briefly, the animals were brought to a central veterinary facility in Antananarivo where local butchers prepared the carcasses. The muscles of the right-hand side of the carcase as well as the masseters, tongue, full diaphragm and heart were dissected from the bodies and sliced by hand at approximately 3mm intervals to reveal all cysts. Where no cysts were identified, the left-hand side of the carcase was assessed for possible infection in a similar way. Cysts were recorded as either viable or non-viable and their numbers were counted to determine the level of infection.

The prevalence of human *T. solium* taeniasis was determined by Kato-Katz thick smear as described in the World Health Organization (WHO) Bench aid for the diagnosis of intestinal procedures [31]. For the baseline evaluation, one smear was prepared for most of the samples, while 2 smears were examined for 78 samples. Confirmation of the *Taenia* species present in Kato-Katz positive samples was performed by PCR and sequencing of a segment of the small subunit of mitochondrial ribosomal RNA (rrnS) gene, as described in Lightowlers et al. [32]. All endline examinations were undertaken on duplicate smears.

### Data analysis

All collected data were entered into Microsoft Office Excel 2013 spreadsheets. Statistical analyses were performed using R 4.3.0 software.

Humans when infected by *Taenia* (i.e., taeniasis), tend to be infected by a single tapeworm and even if infected by several tapeworms, there is no ethically acceptable method to diagnose the intensity of infections (i.e., number of tapeworms in an infected human). It is only possible to detect the presence of infection. Thus, analysis of human infection was most appropriate using a bivariate analysis, i.e., presence or absence of taeniasis, and hence the Chi square test was used. In contrast pigs can be non-infected (no cysts) or infected (1 to thousands of cysts in a pig). Thus, it was possible to statistically analyse not only presence or absence of infection in pigs but also the abundance of infection in infected pigs. The negative binomial model was used because numbers of cysts are count data, and the counts are not Poisson distributed (i.e., over dispersed, the mean number of cysts per pig is much less than the variance of the mean). For the other remaining proportion comparison tests, the chi-square test was used and a p-value of less than 0.05 was considered significant.

## Results

### Initial pig census

The number of pigs in the intervention area was assessed to be 27,071 belonging to 12,006 pig farmers, representing an estimated average of 2 pigs per farm. The majority of farmers allowed their pigs to roam freely. Some pigs went with the farmers to the field during their agricultural activities, while others roam around the villages to search for food. Most of the time, breeding pigs and their piglets, as well as pigs entering the fattening phase, are left free to roam, and some will only be confined in a pigsty at the end of the fattening phase.

### Pig treatments (vaccination and medication)

The initial vaccination and medication of the pig population (from October to November 2021) reached 19,135 pigs for the first immunization, representing 70.68% of the pig population determined by the census prior to the beginning of the pig treatments. For the second immunizations the number of pigs vaccinated decreased to 8,120 of those previously vaccinated (p < 0.001, 42.4% CIs 41.7% – 43.1%). This change was associated with the appearance of unsubstantiated rumors. Efforts were made subsequently to identify, vaccinate and medicate those pigs that missed the second scheduled immunization during the following visits, however some of these animals were typically in the fields and hence unavailable when the vaccinators visited the farms.

**Fig 3:**
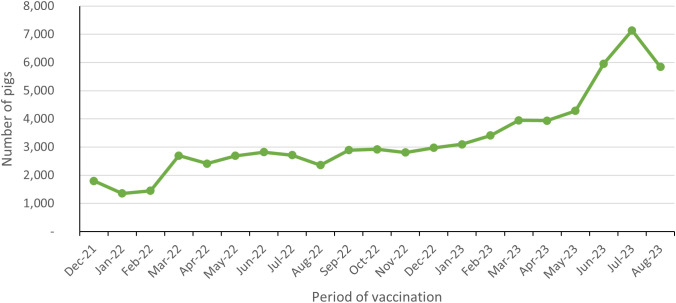
Number of pig vaccination and medications recorded per month during the *T. solium* intervention program. Piglets ≥2 months and any pigs imported into the program area and eligible were targeted for vaccination and medication, twice each, throughout the intervention period.

Pig treatments that subsequently targeted piglets ≥2 months and new imported pigs increased progressively in number throughout the intervention period (Table 1 and Fig 3). On average, 3,309 (SD ± 1,434) treatments per month were carried out. The number of treatments per month increased fourfold between January 2022 and August 2023. The increase in the number of vaccinations (and coverage estimate), was due to additional targeted awareness campaigns (focusing in the areas with lower coverage), and increased vaccinators’ time in the field. In total, 96,735 vaccinations and pig medications were delivered during the program.

**Table 1:** Number of pig treatments, coverage estimate, and number of fokontany achieving >75% coverage estimate.

Month	Number of pig treatments	Coverage estimate	Percentage of fokontany with coverage estimate >75%
Oct – Dec 2021	29,053	NA^*^	NA
January 2022	1,354	NA	NA
February 2022	1,451	NA	NA
March 2022	2,693	NA	NA
April 2022	2,413	NA	NA
May 2022	2,687	NA	NA
June 2022	2,819	NA	NA
July 2022	2,712	NA	NA
August 2022	2,357	71.39%	45%
September 2022	2,891	79.18%	57%
October 2022	2,922	85.48%	73%
November 2022	2,807	77.18%	57%
December 2022	2,973	75.83%	65%
January 2023	3,098	89.34%	71%
February 2023	3,411	89.76%	75%
March 2023	3,946	91.85%	80%
April 2023	3,936	92.84%	83%
May 2023	4,281	95.25%	81%
June 2023	5,954	87.85%	73%
July 2023	7,135	92.54%	82%
August 2023	5,842	93.58%	83%

* NA: not available.

Data on the coverage estimate of vaccination and medication (Table 1) was obtained from vaccinators from August 2022. The coverage estimate is not necessarily aligned with the number of treatments (numerator), as it also depends on the number of eligible pigs identified by the vaccinators (denominator). On average, vaccinators identified approximately 7,000 pigs (eligible and ineligible, vaccinated and medicated, and unvaccinated) per month. Between August 2022 and December 2022 this coverage estimate varied between 71% and 85%; after December 2022, the rate varied between 87% and 95%.

The vaccination and medication coverage in slaughter-age animals was also assessed from the random selection of pigs during the endline evaluation (pigs born approximately September 2022 with scheduled first and second treatments in November and December 2022) where 70 animals (67%, CIs 57% to 76%) among the 104 selected pigs had each received two vaccination and medication treatments. This figure is a close approximation to the coverage estimates for November and December 2022, of 75–77% (Table 1).

The coverage estimate per month varied from one fokontany to another. Detailed records of vaccination and medication coverage estimates show a progressive increase in the percentage of the 84 fokontany achieving a coverage estimate over 75%, with >92% coverage estimate achieved in the final months of the program (Table 1). From August 2022 when proxy coverage estimates were obtained until the end of the year (5 months), 11 fokontany out of the 84 had a coverage estimate of <50% (seven fokontany on one occasion, three fokontany on two occasions, and one fokontany on 3 occasions). In comparison, during the 8 months of 2023, only one fokontany had once a coverage estimate below 50%.

### Human intervention

In total, 117,216 persons were treated during the MDA, which represented 62.52% (CIs 62.30 – 62.74) of the eligible population (187,490 out of 231,755 total population). Significant differences in treatment coverage rates were observed between municipalities (Table 2). Fidirana municipality had the highest number of people treated, with a coverage rate approaching 90%, while Soavina represented the area with the lowest participation, with 40% of the population. The proportion of children under 15 years old participated in the MDA was significantly higher than the proportion of older people that participated in the MDA (p < 0.0001). Among the persons treated, 615 received niclosamide as they were identified as having symptoms or signs compatible with NCC, which represent 0.52% of the treated population. Among persons treated with niclosamide, the proportion of people more than 15 years old was higher than the proportion of children less than 15 years old (p < 0,0001).

**Table 2:** Population treatment data for the taeniacide MDA intervention.

Municipalities areas	Total Population	Children 5–14 years old	People > 15 years old	Children 5–14 years old treated (%)		People > 15 years old treated (%)		MDA coverage (%)
				Praziquantel	Niclosamide	Praziquantel	Niclosamide	
Ambohimasina	26,727	7,350	14,272	4,443 (40.48)	6 (0.05)	6,489 (59.13)	37 (0.34)	10,975(50.76)^a^
Inanantonana	17,012	4,678	9,084	3,357 (33.44)	0	6,681 (66.56)	0	10,038 (72.94)^b^
Ambohimanambola	31,037	8,535	16,574	5,633 (37.12)	7 (0.05)	9,423 (62.09)	113 (0.74)	15,176 (60.44)^c^
Soavina	19,046	5,238	10,171	2,723 (43.92)	1 (0.02)	3,458 (55.77)	18 (0.29)	6,200 (40.24)^d^
Antohobe	17,877	4,917	9,546	3,217 (39.93)	15 (0.19)	4,790 (59.46)	34 (0.42)	8,056 (55.70)^e^
Ankazomiriotra	41,800	11,495	22,322	6,440 (36.17)	34 (0.19)	11,217 (63.00)	113 (0.63)	17,804 (52.65)^f^
Betsohana	12,188	3,352	6,508	2,779 (32.06)	17 (0.20)	5,823 (67.18)	49 (0.57)	8,668 (87.91)^g^
Fidirana	38,308	10,534	20,456	9,641 (34.65)	20 (0.07)	18,101 (65.05)	65 (0.23)	27,827 (89.79)^h^
Vinany	27,760	7,634	14,824	4,205 (33.72)	10 (0.08)	8,181 (65.59)	76 (0.61)	12,472 (55.53)^i^
TOTAL	231,755	63,733	123,757	42,438 (36.20)	110 (0.09)^j^	74,163 (63.27)	505 (0.43)^k^	117,216 (62.52)
				42,548 (66.76)^l^		74,668 (60.33)^m^		

a,b,c,d,e,f,g,h,i: p<0,0001; j vs k: p <0,0001; l vs m: p<0,0001.

There was one case of a severe neurological adverse event. A 13-year-old child among those treated with praziquantel, presented in the evening of the first day of MDA headache, which became stronger during the night; the next day, he vomited and had seizures. This severe adverse event was promptly identified and managed as per established protocols [28]. A CT scan revealed three parenchymal NCC lesions. The child received appropriate medical care, there have been no recurrence of seizures and is leading a normal life.

### Program evaluation—Pigs

For the baseline, 104 pigs were randomly selected as described by Mananjara et al. [30]. At the time of the final evaluations, a census of slaughter-age pigs identified a total of 578 pigs in the fokontany selected for evaluation, including 407 animals (70.4%) that had received 2 treatments (vaccinations and medications), 115 (19.9%) that had received one treatment (one vaccination and medication) and 56 (9.7%) that had received no treatment. From the animals that had received 2 treatments or no treatment, 104 were selected at random for postmortem assessment, which resulted in 70 treated animals (67.3% of the selected animals), and 34 non-treated (32.7% of the selected animals). Pigs that had received only a single vaccination and oxfendazole treatment were not assessed at necropsy. Among the 104 pigs evaluated at baseline, the prevalence of viable *T. solium* infection was 30.77% whereas the prevalence of viable infection in the 104 pigs assessed at final evaluation was 7.69% (Table 3), a statistically significantly difference (p < 0.001). Among the randomly selected slaughter-age pigs at the endline necropsies that had not been vaccinated and treated, 8 out of 34 (23.5%) were found to be infected with viable *T. solium* cysts. Among the vaccinated and medicated animals, no viable *T. solium* cysts were found. Five vaccinated and medicated animals were found to have non-viable lesions in the muscles (4 animals with up to 10 lesions, one with 83 lesions).

**Table 3:** Necropsy results of the baseline and endline program evaluations.

	Baseline*	*Endline*	
Status of pigs	Non treated	Vaccinated + oxfendazole (2 times)	Non treated
Number of pigs	104	70	34
Number with viable cysts	32	0	8
1 - 10 cysts	8	0	6
11 - 100 cysts	8	0	1
101 - 500 cysts	11	0	1
>500 cysts	5	0	0
Number with only non-viable cysts	5	5	1
Number with no cysts	67	65	25
Prevalence with viable cysts % (95% CI)	30.77^c^ (22.08-40.57)	0^a^ (0-5.13)	23.53^b^ (10.74-41.17)
		7.69^d^ (3.37-14.59)	

*, data detailed in [30]; a vs b; a vs c; c vs d p<0,001 c vs b p=0.55.

Among the total of 50 slaughter-age pigs that were evaluated by necropsy at the end of the program, and had not received any vaccination or oxfendazole treatments, that is the 34 pigs randomly selected for the final evaluation plus the additional 16 purposively selected amongst the non-vaccinated or treated pigs, 15 (30%) were found to have *T. solium* cysticerci in their muscles. One had only non-viable cysts. Comparison of the prevalence of infection at baseline and in untreated pigs at endline was not statistically significant (p = 0.6). A significant reduction was observed in the number of *T. solium* cysts (viable or non-viable) in non-vaccinated or treated animals that were evaluated at the final evaluation, compared to the animals assessed at baseline (Table 4, p = 0.0005).

**Table 4:** Reduction in the burden of *T. solium* cysticerci in non-vaccinated or treated pigs. The number of *T. solium* cysticerci (viable and non-viable) was evaluated by necropsy in non-vaccinated or treated pigs before and after the intervention.

Pigs non-vaccinated or treated	Number of pigs evaluated	Number of pigs with cysts	Mean number of *T. solium* cysts in infected animals	Confidence interval
Baseline	104	37	464^a^	226-1195
End of the program ^c^	50	15	26^b^	10-93

^a^vs

^b^p = 0.00005;

^c^includes 34 pigs randomly selected for the final evaluation plus 16 additional non-treated pigs, purposively selected.

### Program evaluation—Humans

Assessment of 960 people purposively selected from the population, Kato-Katz identified taeniasis in 12 persons (1.25%) at baseline [32] and 6 persons (0.6%) one year after the single MDA intervention. The difference was not statistically significant (p = 0.13). Of the total 18 positive fecal samples, 15 (9 from baseline, and 6 from final evaluation) were confirmed as *T. solium* by copro-PCR and DNA sequencing*.*

Among the 960 samples tested at baseline, eleven egg-positive samples were identified to be positive among the samples examined in a single Kato Katz slide except for one, which was identified among the 78 baseline samples tested by 2 slides, and it was positive in both slides. The six samples identified as Kato-Katz positive during the final evaluations were all positive in both slides tested.

## Discussion

The One Health strategy implemented in this program significantly reduced the prevalence of viable porcine cysticercosis over the term of the program. The prevalence of viable *T. solium* infection detected in the 104 animals necropsied at both baseline and endline evaluations was reduced from 30.77% to 7.69% (p < 0.001, Table 3). No infection with viable *T. solium* was identified in the animals that had received two vaccinations and medications as piglets.

Five animals that had received two vaccination and medication treatments were identified with non-viable cysts only. The *T. solium* vaccine targets an antigen uniquely associated with the oncosphere life cycle stage [33] and is not believed to affect cysticerci that may have established in the tissues prior to vaccination. However, the simultaneous treatment with oxfendazole kills any such pre-existing cysts in the muscles [34,35]. For this reason, the five animals detected with only non-viable lesions in vaccinated and treated pigs at the endline necropsies are interpreted as being animals that had been exposed to infection prior to treatment. The important point is that these non-viable lesions present no risk for transmission of *T. solium* infection. The finding that some of the piglets were likely to have been exposed to *T. solium* prior to being vaccinated and treated highlights the importance of delivering the oxfendazole dose in full to the piglets; failure to do so could potentially result in the presence of viable cysts in vaccinated animals (due to infections established prior to vaccination), which could appear as vaccine failures.

A highly significant reduction was observed in the number of *T. solium* cysts (viable or non-viable) in non-treated animals that were evaluated at the final evaluation, compared to the animals assessed at baseline (Table 4, p = 0.0005). This is interpreted as reflecting a reduction in environmental contamination with *T. solium* eggs in the intervention area and a decreased risk of transmission.

There were variations in the coverage estimates of pig treatments, influenced by farming activities which resulted in the farmers leaving the house very early and not being available to facilitate the work of the vaccinators, or by the farmers taking the pigs with them to the fields, such as during the months of December and January which is the rice transplanting season, or harvesting time in June. Throughout the program the coverage estimates of the pig population for treatments steadily increased (Table 1). Soon after the program began, rumours began to circulate that caused many farmers to decline to have their animals vaccinated. For example, it was believed by some that the program was a conspiracy by the government to stop the farmers from breeding local breeds of pigs. It also coincided with a high anti-vaccine general attitude in the population due to the ongoing issues concerning human vaccination for COVID. The farmers rarely vaccinated their animals for any purpose, even though epidemics of Classical Swine Fever cause mass pig mortality and there is a vaccine available, and hence they have a rudimentary understanding of vaccines, if any. Our use of ear tags to indicate treated animals was initially also a source of concern for the farmers as it was not usual. Continuing efforts throughout the program to educate the farmers and dispel the rumours saw the dissipation of negative rumours and a steadily increasing rate of participation. The fact that treated animals were often judged by farmers to be growing faster helped improve the participation rate. This could be due to the effect of the oxfendazole treatment on nematode and other parasitic infections [36,37].

The endline assessment in pigs was undertaken in slaughter age animals, generally around 12 months of age (born approximately August 2022). These animals were raised when coverage of the pig population was 67% (as judged by the randomly selected slaughter-age animals taken for endline necropsy). Hence the decrease in the prevalence of viable *T. solium* infection in the pig population (30.8% to 7.7%) was achieved in animals born when the coverage of the eligible pig population with two vaccinations and medications was also approximately 70% (Table 1). Correspondence between the treatment coverage estimate among animals born approximately 1 year before the sampling of animals for necropsy, and the proportion of slaughter-age animals which had received two vaccination and medication treatments identified at the time of the endline necropsies, provides a degree of confidence about the accuracy of the figures obtained as treatment coverage estimates (Table 1). Towards the end of the program, treatment coverage estimates for piglets receiving two vaccinations and medications were approximately 90%. It could be expected that the program may have achieved a greater reduction in transmission through pigs had it been possible to undertake necropsies on slaughter-age animals that had been born later in the program when the piglet coverage estimate had increased. A program of continued vaccination and medication of piglets and animals imported into the program area, would maintain control of *T. solium* transmission indefinitely. In Madagascar it may be possible to extend the program to the entire country.

The intervention program met a major challenge with implementing the planned MDA in humans. The plan was to undertake the MDA a short time after the entire pig population had been vaccinated and medicated, and piglet vaccination and medications were well established and continuing. However, a serious adverse event in 2021 during the routine Malagasy schistosomiasis MDA program with praziquantel resulted in a child’s death in a district nearby the program area, delaying the MDA of this program until further measures were put in place. This situation also had an impact on the population’s participation in the program’s MDA. The population coverage for the MDA was 62.5% while the objective was to reach >75% of the population. In addition, the treatment coverage in the pig population was less than 90%. This being the case, it was not surprising to find that *T. solium* taeniasis remained present in the population at the endline assessments and transmission continued to non-treated pigs (as evidenced by the rate of infection found in non-vaccinated or medicated pigs). An earlier taeniasis MDA program in Madagascar, in a nearby area undertaken prior to the occurrence of the aforementioned adverse event achieved a 95% coverage [9] and an MDA with praziquantel in the study area population conducted approximately 6 months after the final evaluation of this program achieved 82.9% coverage [28], suggesting that the population is not averse to participating in MDA programs. Had there been more time between the adverse event that occurred during the schistosomiasis program and our MDA, a higher coverage of the population may have been achieved. There were also significant differences in the initial human MDA coverage in the different fokontany in the study area, ranging from 40% to 90% (Table 2). Because of the low prevalence of taeniasis in the baseline survey, it was not possible to analyse if this heterogeneity in coverage had any effect on outcome of the study or of any effect on groups at risk.

An unanticipated but valuable outcome of our program has been the development of protocols, resources and a strategy to minimize the potential for adverse events occurring due to MDA being undertaken with praziquantel in an area that is, or may be, endemic for *T. solium*. These resources are available through the WHO [38,39] and the measures are discussed in full by Nely et al. [28].

A feature of this *T. solium* control program was that it was implemented not as a research project, but as a public health program, carried out by the two responsible ministries in Madagascar (Ministry of Public Health and Ministry of Livestock). In this way, it was implemented by existing and already responsible structures. It was provided to all the people in the control region, without cost. Funding was provided from the program’s financial sponsors to the Ministries, which either funded their staff time, or was distributed to their contractors, who undertook the work. Existing community agents and community health workers participated in the human MDA-associated activities, and local veterinarians and their associated vaccinators conducted the work on the animal side. Community animal health workers are present in many African, Asian and Latin American countries [40,41] and could potentially facilitate vaccination and medication of pigs such as those that were undertaken in Madagascar. The involvement of the Ministries was planned with a view to appropriation and sustainability, as well as extension once the program was completed and demonstrated to be successful. While the program was implemented as a public health program, additional resources were provided specifically so as to provide a rigorous evaluation of the program’s outcomes. An additional outcome of the program, was an improved relationship between the Ministries and other relevant stakeholders, reflecting the benefits of strengthening the One Health systems for future collaborations and preparedness for future challenges.

The program was designed to require what was considered to be minimum inputs and to have a rapid impact, so as to maximize the feasibility of the program being implemented in other *T. solium* endemic regions of Madagascar, and possibly elsewhere. By minimum inputs we refer to only one MDA and one intervention in the whole pig population, followed only by interventions in piglets and incoming new pigs. As the period available for the entire program was limited by the funder to two years, it was decided to initiate the intervention by vaccinating and oxfendazole treating the entire eligible pig population. A potential advantage of that approach was an anticipated rapid impact on porcine cysticercosis and hence the incidence of human *T. solium* taeniasis. An alternative approach in the intermediate hosts, would have been to rely on piglet interventions (and of imported animals), thereby avoiding the initial treatments to the entire pig population, followed by an MDA after treated animals had largely replaced the adult pig population. Although simpler, such a program would take longer to impact human-to-human transmission of *T. solium* while the existing population of infected pigs gradually left the population through normal slaughter practices or natural attrition. The program’s reliance principally on treatments in piglets has the advantage that young animals are much easier to locate, catch and handle, compared to directing treatments to more mature pigs. As an on-going program, directing the treatments to piglets avoids the potential for medicated animals to be slaughtered within the drug withholding period and the potential for the drug treatment to render carcases inedible because of the presence of drug-killed necrotic cysticerci in the meat.

The program provided MDA treatment drugs through a donation via the WHO and the program funding supported the cost of the vaccine, oxfendazole and vaccinators who delivered treatments to the animals. While we consider that the intervention measures used here represent a minimum inputs approach, which had a major impact on the transmission or *T. solium* through pigs, we acknowledge that without the resources from external donors it is unlikely that control of this neglected zoonosis could be undertaken in Madagascar, or elsewhere, where full transmission of the parasite occurs.

**Fig 4:**
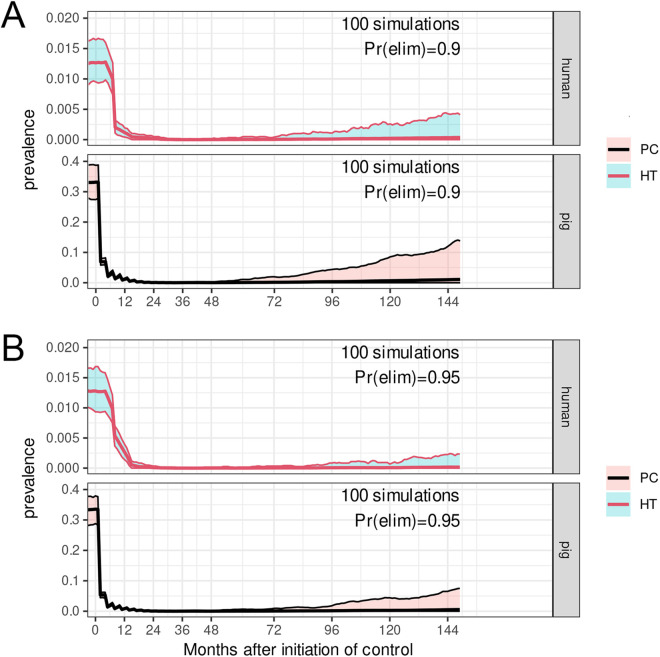
Modelling of the impact of an 18-month *T. solium* intervention involving pigs and humans. **(A)** Pig vaccination and oxfendazole medication covering 80% of slaughter-age animals and a single MDA of 80% of the human population with praziquantel 6 months after the implementation of pig treatment predicts a 90% likelihood of elimination of *T. solium* transmission. **(B)** Pig vaccination and oxfendazole medication covering 85% of slaughter-age animals and a single MDA of 40% of the human population with praziquantel 6 months after the implementation of pig treatment predicts a 95% likelihood of elimination of *T. solium* transmission. (PC: porcine cysticercosis, HT: human *T. solium* taeniasis; Pr(elim): probability of elimination of transmission. CystiSim [18]).

The public health program implemented here achieved a reduction in the prevalence of porcine cysticercosis from 30.8% to 7.7% with viable infections only being identified in animals that did not receive the two vaccinations and oxfendazole treatments as piglets. The program achieved a 90% estimated coverage of the piglet population within 20 months of the start of piglet vaccination. This reduction may be sustainable within the project area through a continuation of the treatments in piglets and expansion of the program to neighboring endemic areas. People from endemic areas in which control is not implemented could have taeniasis and come into the program areas, but if a sufficiently high proportion of the pigs in the program area were protected with the vaccine, the impact would be minimum as the transmission cycle could not be maintained. In the long term, if this happened after the pig vaccination was stopped, their effect would depend on the persistence of the necessary risk factors for the parasite’s transmission cycle to be maintained. Modelling by CystiSim [18] predicts that a similar program to that implemented in Madagascar, implemented over an eighteen-month period and achieving an 80% coverage of the pig population together with a single MDA of the human population 6 months after pig treatments were established, would achieve the on-going elimination of *T. solium* transmission in the absence of parasite re-introduction into the control area (Fig 4A). Modelling of transmission where treatment of pigs achieved 85% of population coverage and a single MDA program achieved a 40% coverage of the population, predicts a 95% probability of elimination of transmission (Fig 4B), indicating the critical nature of control activities in pigs in achieving reductions in *T. solium* transmission.

The program had several limitations. We were unable to undertake an assessment of *T. solium* infection in pigs that were born towards the end of the 20-month study, when the highest levels of population coverage by vaccination and medication had been achieved. The period required to secure a high level of participation in the program by farmers, and a limit on the duration of the project by the funder, precluded the MDA being delivered at the optimum time (when the pig treatment rate was > 80%). The sample size used for assessment of human *T. solium* taeniasis was insufficient to determine whether a statistically significant reduction in taeniasis had been achieved by the program. Pigs that received only a single vaccination and medication treatment were not included in the final necropsy assessments, although the numbers of non-treated animals necropsied reflected the proportion of the total slaughter-age population that either had received no treatments or had received only a single treatment, which might underestimate the efficacy of the program, as a single dose of oxfendazole may have had some positive effect.

In conclusion, this minimum input One Health *T. solium* control program significantly reduced parasite transmission in the intervention area over a period of 20 months. The intervention was delivered as a public health program and therefore reflected the real-life challenges when implementing these types of programs in the field, providing a possible model for future *T. solium* control programs.
